# Parental alcohol misuse and hazardous drinking among offspring in a general teenage population: gender-specific findings from the Young-HUNT 3 study

**DOI:** 10.1186/1471-2458-13-1140

**Published:** 2013-12-06

**Authors:** Siri H Haugland, Turid L Holmen, Edle Ravndal, Grete H Bratberg

**Affiliations:** 1 HUNT Research Center, Department of Public Health and General Practice, Faculty of Medicine, Norwegian, University of Science and Technology, Forskningsveien 2, Levanger 7600, Norway; 2 Department of Research and Development, Levanger Hospital, Health Trust Nord-Trøndelag, Levanger 7600, Norway; 3 Norwegian Centre for Addiction Research, University of Oslo, P.O. Box 1039, Oslo, Blindern 0315, Norway

**Keywords:** Adolescence, Parents, Alcohol consumption, Alcohol misuse, Gender, Generations, Family, Environment

## Abstract

**Background:**

Parental alcohol misuse may negatively affect drinking behaviours among offspring, but it is unclear to what extent influences are gender-specific and dependent upon the actual drinking behaviour measured. The aim of this study was to investigate whether hazardous drinking among Norwegian teenage boys (N = 2538) and girls (N = 2494) was associated with paternal and maternal alcohol misuse (CAGE).

**Methods:**

Definitions of hazardous drinking among offspring were based on self-reported alcohol consumption (in litres a year), frequency of drinking, and frequency of drunkenness. Based on this information, two composite measures of hazardous drinking were also constructed. Cross-sectional data from the Norwegian Young-HUNT 3 survey (2006–2008) were linked to information from biological parents who participated in the adult part of the HUNT study.

**Results:**

Logistic regression analyses showed that both boys and girls with alcohol misusing fathers were more likely to report high levels of alcohol intake compared to others of the same age and gender. This was contrary to boys with misusing mothers, who reported less alcohol consumption than other boys. Among girls, but not boys, high frequency of drunkenness was associated with maternal as well as paternal misuse.

**Conclusions:**

This study suggests that adolescent hazardous drinking is more prevalent among boys and girls with alcohol misusing parents versus those whose parents do not misuse alcohol. However, findings were gender specific and varied depending on the drinking outcomes under investigation. More evidence-based knowledge in this field is of great importance for better understanding the possible role paternal and maternal alcohol misuse may play in the development of hazardous alcohol drinking patterns among adolescent boys and girls.

## Background

Harmful alcohol use is a major contributor to the global burden of disease, premature deaths, disabilities, and neuropsychiatric disorders. Alcohol misuse can be related to both chronic disease and acute injury, in addition to social, psychological, and economical harm [1-4]. Initiation of alcohol use during adolescence is prevalent both in Norway and in many Western countries [5,6]. The 2011 ESPAD report demonstrates that, even though Norwegian adolescents reported less drug and alcohol use than adolescents in the majority of the participating European countries, the average amount of alcohol consumed during the most recent drinking day was among the top three highest levels [7]. Alcohol is also commonly used among Norwegian adults [8].

Alcohol epidemiology has traditionally focused on consumption volume, but in recent years drinking patterns such as frequent drinking and heavy episodic drinking (including binge drinking and intoxication) have gained increased attention [9]. Hazardous drinking can be defined as a pattern of alcohol use that increases the risk of harmful consequences for the user [10]. Volume of consumption has been associated with numerous diseases in a dose–response relationship [3,4,11]. Other studies found that frequent drinking at 14–15 years of age predicts alcohol dependence at ages 20–21 [12], while heavy episodic drinking typically has been related to short-term acute injuries that may be fatal [13].

Hazardous drinking becomes more prevalent with age during adolescence. Several factors may increase the likelihood of hazardous alcohol consumption, such as hereditary predisposition, impaired parenthood, parental divorce, social deprivation, risky use among peers, early pubertal timing, and mental health and behavioural problems [14-19].

A history of alcohol misuse in the family may also increase the risk of hazardous drinking in adolescence [18,20-25]. WHO’s European regional office estimated in 1998 that 4.5 million young people in the EU region lived in families adversely affected by alcohol [26]. In Norway it has been estimated that 8.3% of children live in homes where at least one of the parents suffer from an alcohol use disorder [27].

Although there is well-established research regarding the effects that familial alcohol use disorder has on offspring’s risk of problematic drinking, a large part of the studies on children with parents who abuse alcohol has relied on clinical samples. This may have led to an overestimation of adverse consequences due to the selection of more severely impaired parents [24]. Using population-based samples may contribute to expanding existing knowledge about the consequences of parental alcohol misuse on offspring drinking patterns in the general population.

It is unclear whether parental and offspring gender may be of importance for the association between parental and offspring alcohol use, and findings of possible gender effects have been inconclusive [28-32]. Lieb et al. [28] found that the impact of parental alcohol use disorders was comparable for male and female offspring, but consequences depended on the gender of the misusing parent. While maternal alcohol misuse was associated with a higher risk of progression from occasional to regular use, paternal alcohol misuse was associated with progression from regular to hazardous use. Zwaluw et al. [33] found that maternal, but not paternal, problem drinking was associated with alcohol use among the oldest adolescents (16–17 years of age). Paternal problem drinking was, however, associated with alcohol use in younger adolescents (14–15 years of age). A recent study [34] found that female college students with alcohol misusing mothers had greater risk of problematic alcohol consumption compared to those with alcohol misusing fathers.

Other studies have failed to prove gender as an important factor in response to parental alcohol problems [35]. Some of the inconsistency in gender-specific findings regarding parental alcohol misuse and gender might be due to the great variety of study designs, sample sizes, and measurements, making it difficult to compare findings across studies. Another challenge for comparisons is that drinking patterns largely differ between countries and cultures. Studies including both parental gender and offspring gender are needed for a better gender-specific understanding of the relationship between parental alcohol misuse and hazardous drinking among adolescent offspring [36].

The aims of this study were to investigate whether hazardous drinking in a sample from the general population of adolescents at 13–19 years of age was associated with biological mother’s and/or father’s alcohol misuse, and whether possible associations were related to offspring gender.

## Methods

### Design and participants

This study combines data from adolescents and their biological parents who participated in the youth or adult part of the third wave of the Nord-Trøndelag Health Study (Young-HUNT 3 or HUNT 3, 2006–2008). All inhabitants aged 13 years or older and living in Nord-Trøndelag (the central Norwegian county with approximately 127,000 inhabitants) were invited to participate. Adult participants completed self-report questionnaires (somatic and mental health, lifestyle, quality of life, use of medication, use of health services) and attended clinical examinations^a^. Parental alcohol consumption was reported in two different questionnaires (Q1 and Q2), where alcohol misuse (CAGE) was included in Q2. The response rate for Q1 was 54% (N = 50807), of whom about 80% responded to Q2 (i.e. 27,758 women and 23,049 men). For more information about the HUNT 3 survey, see Krokstad et al. [37].

The youth part of HUNT 3 (Young-HUNT 3) was conducted in school settings, and the students completed a self-administered questionnaire during one school hour. All students in junior and senior high schools, aged 13–19 years, were invited to participate. Adolescents outside the school system were identified through lists obtained from the local authorities. In the Young-HUNT3 survey, 8200 participants (78% response rate) completed the questionnaire [38], where 115 of the participants were obtained outside the school system [38]. In this study we included 5032 (61%) students who could be linked to either biological mother’s and/or father’s responses (questionnaire 2) in the HUNT 3 survey. Through the Norwegian Family Register, using the 11-digit personal number given to every Norwegian at birth, it was possible to merge data between 4174 adolescents and their biological mothers, and between 3123 adolescents and their biological fathers. A higher proportion of the adolescents had data from mothers compared to fathers (83% vs. 62%). Nearly half (45%) of the study population had information from both biological parents (Table 1). An equal proportion of boys (n = 2538) and girls (n = 2494) were included.

**Table 1:** Characteristics and descriptive statistics of the adolescents and parents included in the study sample, by gender

	**Total**		**Fathers**		**Mothers**	
			**misuse**	**No misuse**	**misuse**	**No misuse**
	**n**	**%**	**%**	**%**	**%**	**%**
All	5032	100				
Boys	2538	50.4				
Girls	2494	49.6				
Data from father	3123	62.1				
Data from mother	4174	82.9				
Data from both parents	2265	45.0				
Parents divorced/separated	1114	24.4	22.8	17.2	36.9	22
Education level, fathers						
*Low* (NSCE* group 0–2)	627	14.2	13.5	12.4		
*Medium* (NSCE* group 3–5)	2628	59.5	60.9	59.1		
*High* (NSCE* group 6–9)	1162	26.3	25.6	28.5		
Education level, mothers						
*Low* (NSCE* group 0–2)	603	12.0			11.2	11.6
*Medium* (NSCE* group 3–5)	2261	46.8			39.3	47.3
*High* (NSCE* group 6–9)	1969	40.7			49.4	41.2
Alcohol misuse, fathers	468	15.6				
Alcohol misuse, mothers	181	4.7				

*Norwegian Standard Classification of Education.

When considering the possibilities of selection bias, we made some comparisons between those included (with parental data) and those excluded (without parental data). We found no statistically significant differences in the prevalence of hazardous drinking behaviour between the groups, but parental divorce was more common among those excluded than among those included (24.4% vs. 35.5%, p < .001), and those excluded were also on average slightly younger (15.8 years vs. 15.9 years, p = 0.047). Details are included in Additional file 1.

### Measures

Measuring alcohol consumption patterns among adolescents entailed questions about frequency of drinking, number of intoxication events, and beverage-specific alcohol consumption.

As alcohol consumption becomes more prevalent with age, we used age dependent cutoffs (i.e. relative to peers of same age) to define frequent drinking, frequent intoxication and high level of alcohol consumption.

Students who initially reported never consuming alcohol or not knowing if they had ever tried alcohol were asked to pass additional questions about alcohol use. Missing data due to this initial question was recorded as “no use” in the dataset, but if respondents reported alcohol use in later parts of the questionnaire, the latter response was held as valid.

#### Frequent drinking

Students were asked “How often do you usually drink alcohol”, with five response categories from never to every week or more often. Students in lower secondary school who drank alcohol “every other week or more often” and students in upper secondary school who “drank every week or more often” were defined as *frequent drinkers* (coded 1). Other responses were coded 0.

#### Frequent intoxication episodes

The number of lifetime alcohol intoxication episodes had five response alternatives: Never, once, 2–3 times, 4–10 times, 11–25 times, or more than 25 times.

Any intoxication episode reported by 13–14 year olds, four episodes or more reported by 15 year olds, ten episodes or more reported by 16 year olds, and 25 episodes or more episodes reported by 17–19 year olds were classified as *frequent intoxication episodes* (coded 1). Other responses were coded 0.

#### High levels of alcohol consumption

Information about alcohol consumption was based on students’ reports of how much beer, wine, and liquor they usually drank during a period of two weeks, calculated into litres of pure alcohol a year. Adolescents with consumption above the 75th percentile within each age and gender group were coded 1; others were coded 0.

#### Composite measurement of hazardous drinking

Based on the underlying assumption that adolescents who report several types of hazardous alcohol behaviours are at greater risk of harm than adolescents who report fewer or none, we constructed a composite “hazardous drinking” variable consisting of the three items described above (frequent drinking, frequent intoxication episodes, and/or high consumption levels), with a maximum score of 3. A score of 1 was categorized as “moderately hazardous” and 2 or 3 were categorized “highly hazardous”. In multivariate analyses, moderately and highly hazardous drinkers were compared separately with those without any of the hazardous drinking behaviours (coded 0).

#### Parental alcohol misuse

Parental alcohol misuse was measured by the four items of the CAGE questionnaire [39]. CAGE is one of the most widely validated screening tools for detecting alcohol abuse and dependence in primary care. Standard CAGE consists of four items: 1) Have you ever felt that you ought to **C**ut down on your drinking? 2) Have people **A**nnoyed you by criticizing your drinking?^b^ 3) Have you ever felt bad or **G**uilty about your drinking? 4) Have you ever had a drink first thing in the morning to steady your nerves or to get rid of a hangover (**E**ye-opener)? CAGE has demonstrated high test–retest reliability (0.80–0.95) [40]. Skogen et al. [41] examined the concurrent validity of the CAGE questionnaire in previous HUNT studies and found the internal validity of CAGE to be adequate (Kuder Richardson 0.68). They also found a linear relationship between both current and previous excessive alcohol consumption.

The standardized and recommended cut-point for the CAGE instrument to screen for alcohol abuse and dependence was applied [40]. CAGE scores of ≥ 2 was coded 1 (misuse); those below 2 were coded 0 (no misuse). Parents who reported abstention in Q1 combined with not answering any of the four CAGE questions (Q2) were coded 0 (no misuse). In order to include those with incomplete data (i.e. 1–3 CAGE items), missing data on one or more CAGE items were coded 0. Since CAGE is not a diagnostic tool, the term “misuse” was used for respondents with a score of 2 or higher.

#### Confounders

Parental education, parental divorce/separation, parental depression/anxiety, and adolescent age at screening point were considered as possible confounders [42-46]. Data on parental educational status (year 2008) was retrieved from the Norwegian education register (Statistics Norway) and linked to the data.

*Educational status* was categorized into nine levels of education according to the Norwegian Standard Classification of Education/NSCE [47] and used as a continuous variable in multivariate analyses. Information about *parental divorce/separation* was based on adolescent self-reports that parents had been divorced or separated for more than one year. *Adolescent age* was included as a continuous variable in the multivariate analyses. *Parental depression/anxiety* was measured through parental report using the Hospital Anxiety and Depression Scale (HADS) [48].

### Statistical procedure

Initial descriptive analyses were performed for obtaining an overview of the data. The main dependent variable in this study was “hazardous alcohol drinking”, defined as frequent drinking, frequent intoxication episodes, or high consumption levels, in addition to the composite measures *moderately* or *highly* hazardous drinking behaviours. To investigate if hazardous alcohol consumption was more common among boys and girls with alcohol misusing parents (mothers or fathers) compared to others of the same age, we used multivariate logistic regression analyses. Results were reported as odds ratios (ORs) with 95% confidence intervals (CIs) and with the respective p-values, with a significance level set to 0.05 (*P*). Since the initial analyses revealed statistically significant interactions across genders, analyses were stratified and conducted separately for boys and girls for each of their parents. In the multivariate regression models, odds ratios were adjusted for the possible confounding of adolescent’s age, parental marital status, and education level.

### Ethics

All participants, and in addition the guardians of participants under the age of 16, gave their written consent. The study was approved by the Norwegian Data Inspectorate and the Regional Committee for Medical Research Ethics (REK).

## Results

### Descriptive statistics

In this study 2538 boys and 2494 girls with data from one or both parents were included (Table 1). About one fourth of the respondents reported that their parents had been divorced/separated for more than one year, and among these respondents, half cohabited mostly with their mother, while 37% cohabited equally with both parents or mostly with their father (13%). As Table 1 illustrates, more mothers than fathers had a higher education (41% vs. 26%), and paternal alcohol misuse was more common (16%) than maternal misuse (5%).

### Frequency of hazardous drinking behaviours by status of parental alcohol misuse

Table 2 shows that the frequency (%) of the defined hazardous drinking behaviours differs to some extent between each of the behaviours, but that proportions were fairly similar for both genders. Relatively more boys than girls were classified as frequent drinkers.

**Table 2:** The number and proportion of various hazardous drinking behaviours among boys and girls by status of paternal and maternal alcohol misuse

**Hazardous drinking behaviours**		**Total**		**Fathers**				**Mothers**			
				**Misuse**		**No misuse**		**Misuse**		**No misuse**	
		**n (total)**	**%**	**n (total)**	**%**	**n (total)**	**%**	**n (total)**	**%**	**n (total)**	**%**
**High alcohol consumption**	Boys	479 (2371)	20.2	49 (192)	25.5	232 (1248)	18.6	7 (81)	8.6	362 (1754)	20.6
	Girls	496 (2416)	20.5	62 (257)	24.1	212 (1174)	18.1	26 (90)	28.9	371 (1765)	21
**Frequent intoxication**	Boys	608 (1974)	30.8	53 (152)	34.9	302 (1019)	29.6	14 (64)	21.9	453 (1482)	30.6
	Girls	590 (2162)	27.3	77 (234)	32.9	274 (1063)	25.8	28 (75)	37.3	1029 (1482)	27.6
**Frequent drinking**	Boys	261 (1981)	13.2	31 (156)	19.9	128 (1020)	12.5	7 (64)	10.9	195 (1486)	13.1
	Girls	207 (2161)	9.6	19 (233)	8.2	95 (1066)	8.9	9 (74)	12.2	152 (1581)	9.6
**Hazardous drinking, moderate**	Boys	341 (2055)	16.6	26 (154)	17.9	169 (1087)	15.5	9 (77)	11.7	271 (1531)	17.7
	Girls	392 (2138)	18.3	57 (222)	25.7	182 (1050)	14.9	18 (75)	19.1	289 (1556)	15.7
**Hazardous drinking, high**	Boys	414 (2128)	19.5	44 (172)	25.6	204 (1122)	18.2	9 (77)	11.7	304 (1564)	19.4
	Girls	384 (2130)	18	42 (207)	20.3	170 (1038)	16.4	19 (76)	25	288 (1555)	18.5

### Adolescent hazardous drinking associated with parental alcohol misuse

Tables 3 and 4 reveal the crude and multivariate adjusted odds ratios (ORs) of hazardous alcohol drinking behaviours among adolescents with alcohol misusing parents compared to those without misusing parents (reference group), stratified by both parental as well as offspring gender.

**Table 3:** The odds ratios (ORs, 95% CIs) of various hazardous drinking behaviours among girls and boys by status of paternal and maternal alcohol misuse

			**Girls**						**Boys**			
**Outcomes**	**Exposures**		N*	**OR**	**CI**	***P***	**Exposures**		N*	**OR**	**CI**	***P***
**High alcohol consumption**	Mother misuse	Crude	1855	1.5	1.0–2.4	.078	Mother misuse	Crude	1835	0.4	0.2–0.8	.011
		Adj.	1714	1.3	0.8–2.3	.280		Adj.	1662	0.2	0.1–0.6	.005
	Father misuse	Crude	1431	1.4	1.0–2.0	.026	Father misuse	Crude	1440	1.5	1.1–2.1	.025
		Adj.	1341	1.5	1.1–2.1	.020		Adj.	1336	1.6	1.1–2.3	.018
**Frequent intoxication**	Mother misuse	Crude	1656	1.6	1.0–2.5	.068	Mother misuse	Crude	1546	0.6	0.3–1.2	.141
		Adj.	1528	1.8	1.0–3.1	.035		Adj.	1395	0.6	0.3–1.1	.110
	Father misuse	Crude	1297	1.4	1.0–1.9	.027	Father misuse	Crude	1171	1.3	0.9–1.8	.191
		Adj.	1221	1.5	1.1–2.1	.020		Adj.	1093	1.4	1.0–2.1	.087
**Frequent drinking**	Mother misuse	Crude	1655	1.3	0.6–2.7	.471	Mother misuse	Crude	1550	0.8	0.4–1.8	.612
		Adj.	1528	1.3	0.6–2.9	.454		Adj.	1402	1.0	0.4–2.3	.963
	Father misuse	Crude	1299	0.9	0.5–1.5	.711	Father misuse	Crude	1176	1.7	1.1–2.7	.014
		Adj.	1233	0.9	0.5–1.5	.665		Adj.	1098	1.6	1.0–2.5	.059

*N corresponds to the number of adolescents included in the analyses.

**Table 4:** The odds ratios (ORs, 95% CI’s) of moderate and high hazardous drinking (composite measure) among girls and boys by status of paternal and maternal alcohol misuse

			**Girls**						**Boys**			
**Outcomes**	**Exposures**		N*	**OR**	**CI**	***P***	**Exposures**		N*	**OR**	**CI**	***P***
**Hazardous drinking, moderate**	Mother misuse	Crude	1631	1.4	0.8–2.4	.242	Mother misuse	Crude	1608	0.6	0.3–1.2	.179
		Adj.	1509	1.4	0.8–2.7	.255		Adj.	1448	0.5	0.2–1.2	.138
	Father misuse	Crude	1272	1.6	1.2–2.3	.004	Father misuse	Crude	1241	1.1	0.7–1.7	.670
		Adj.	1200	1.9	1.3–2.8	.001		Adj.	1147	1.3	0.8–2.1	.331
**Hazardous drinking,high**	Mother misuse	Crude	1631	1.5	0.9–2.5	.161	Mother misuse	Crude	1641	0.5	0.3–1.1	.096
		Adj.	1500	1.5	0.8–2.7	.211		Adj.	1479	0.5	0.2–1.1	.103
	Father misuse	Crude	1245	1.3	0.9–1.9	.172	Father misuse	Crude	1294	1.5	1.1–2.4	.022
		Adj.	1167	1.5	1.0–2.2	.077		Adj.	1196	1.7	1.1–2.5	.016

*N corresponds to the number of adolescents included in the analyses.

#### High levels of alcohol consumption

Alcohol consumption levels above the 75th percentile for each age group and gender were more common among boys (OR = 1.6; CI = 1.1–2.3) and girls (OR = 1.5; CI = 1.1–2.1) with alcohol misusing fathers versus those offspring with non-misusing fathers (Table 3, adjusted model). High levels of alcohol consumption were less common among boys with alcohol misusing mothers (OR = 0.2; CI = 0.1–0.6) compared to other boys.

#### Frequent intoxication episodes

Girls with maternal (OR = 1.8; CI = 1.0–3.1) or paternal (OR = 1.5; CI = 1.1–2.1) alcohol misuse were more likely than other girls to report high frequency of intoxication (Table 3). Frequent intoxication episodes in boys were not associated with either maternal or paternal alcohol misuse.

#### Frequent drinking

Frequent drinking among boys was associated with paternal alcohol misuse in bivariate analyses, but in multivariate adjusted analyses this association was no longer statistically significant (Table 3). Frequent drinking among girls was not associated with parental alcohol misuse.

#### Composite hazardous drinking measurement

Moderately hazardous drinking was associated with paternal alcohol misuse for girls (OR = 1.9; CI = 1.3–2.8), but not for boys (Table 4). Highly hazardous drinking was associated with paternal alcohol misuse for boys (OR = 1.7; CI = 1.2–2.5), but not for girls (Table 4).

#### Confounders

High parental education was in most models significantly associated with a reduction in the OR of adolescent hazardous drinking, while divorce/separation and adolescent age increased the OR of hazardous drinking. The associations between the confounding variables and different outcomes varied to some extent depending on both parental and offspring gender. Details on this are included as supplementary material in Tables S6 and S7 found in the Additional file 1. Initial analyses included the Hospital Anxiety and Depression Scale as a possible confounder (HADS-A and HADS-D). Since we found very little evidence for an association between parental anxiety and depression and the different outcomes under investigation, HADS was excluded in the multivariate models.

## Discussion

Our findings verify previous studies showing that alcohol misuse among parents increases the likelihood of hazardous drinking among offspring [21,28,49]. However, results varied depending on gender and the actual drinking behaviours investigated. High levels of alcohol consumption were more common among both boys and girls when their fathers misused alcohol, while it was a tendency that boys with misusing mothers reported less hazardous drinking, but these associations were statistically significant only for high alcohol consumption. Boys were more likely to report highly hazardous drinking (composite measurement) and girls moderately hazardous drinking when fathers misused alcohol. A high number of intoxication episodes were associated with both maternal and paternal alcohol misuse in girls, but not in boys.

### Genetic and environmental factors

Both genetic and environmental factors have been found to be important for the etiology of alcohol consumption behaviours in adolescence [25,50,51]. While alcohol dependence in adulthood has been shown to have a strong heritable component, shared environmental influences seem to have a relatively stronger effect in youth samples and at earlier stages of alcohol use [25]. Throughout adolescence genetic factors gain importance [25] and may explain some of the variation in risky adolescent drinking. A recent Swedish adoption study [52] concludes that drug abuse is an etiologically complex syndrome influenced by a diverse set of genetic risk factors and by a range of environmental factors, along with the interactions between these.

Parenting behaviour is considered to be an important environmental factor that partly explains the relationship between parental alcohol misuse and risky adolescent drinking. Parents who misuse alcohol have been found to provide less emotional support [53], have more problems with maintaining alcohol-specific behavioural control [33], and provide less monitoring [54]. Low levels of monitoring may facilitate more opportunities for offspring to establish risky drinking patterns. Other studies have failed to find parenting behaviours associated with parental problem drinking [33].

Social learning theory [55] offers modelling as another possible explanation for transmission of drinking behaviour across generations. A systematic review from 2010 [56] examined longitudinal studies of parenting factors associated with alcohol consumption in adolescents. Although results varied depending on age and gender, they found that children who had observed their parents consuming alcohol drank more as adolescents than those who had not. In a previous follow-up study of 13- to 14-year-old adolescents, we found that boys and girls who ever had observed their parents drunk also were more likely to report more hazardous drinking four years later [57].

### Aversive transmission

Haller and Chassin [58] found that even though parental alcohol misuse mainly increased offspring drinking, there was evidence for aversive transmission. In accordance with that, we found that boys with misusing mothers were less likely than other boys to report high levels of alcohol consumption.

One possible mechanism for explaining aversive transmission is that offspring with parents who misuse alcohol may reduce their drinking due to their own self-perception of being at risk of future alcoholism [58]. Why maternal (and not paternal) alcohol misuse in our study was associated with decreased risk of high levels of alcohol consumption in boys and not in girls remains unexplained, but this should be a subject for further investigation.

### Gender-specific findings

Research on possible consequences of having parents that misuse alcohol reveals heterogeneity in adverse outcomes associated with both parental gender and offspring gender [42,59-62]. Growing up with parents who misuse alcohol may have a different impact on boys and girls due to influences of both risk and protective environmental factors provided through culture, gender roles, education levels, and other factors. In our study, we found that parental alcohol misuse was associated with hazardous drinking behaviours among adolescents, but associations depended on both parental and offspring gender, as well as the outcomes under investigation.

When addressing gender-specific findings, several questions may arise. Does paternal alcohol misuse represent another kind of “threat” to offspring than maternal misuse, and are there gender-specific susceptibilities in children’s responses to these exposures? Women are, for instance, more likely to use alcohol to self-medicate to cope with problems or traumas in their lives [42], and it has been suggested that maternal misusers exert negative influences of misuse to the child through emotional unavailability [63]. Paternal alcohol misuse, on the other hand, may influence their offspring negatively through other mechanisms, like more prevalent use of violence [42]. Lieb et al. [28] also suggest that fathers probably model more excessive drinking than mothers.

Possible mechanisms between parental and offspring alcohol consumption were beyond the scope of our study, but findings may suggest that alcohol misuse among fathers is more influential than it is among mothers, and that girls may be more susceptible to parental alcohol misuse than boys. Parental alcohol misuse was more often associated with various types of hazardous drinking behaviours in girls than in boys, while hazardous drinking behaviours among offspring more frequently were associated with paternal than maternal alcohol misuse. These differences could be random, but they are nonetheless interesting and may generate hypotheses about gender-specific differences that should be emphasized in future research.

### Limitations and strengths

#### Limitations

Since this study was cross-sectional, any interpretations of causality should be avoided. However, findings based on a large general population of teenage boys and girls as well as their biological parents may generate hypotheses about causal pathways.

There are several limitations associated with self-reported alcohol use and misuse that may have led to inaccuracy in measurements and underestimation of the associations between parental alcohol misuse and hazardous drinking among offspring. First, alcohol misusers tend to be underrepresented in surveys like this. Both heavy alcohol consumption and mental distress have been found to be moderately associated with non-response among adults [64]. In supplementary analyses of adolescents included in this study (with parental data), versus those excluded (without parental data), we found no differences related to hazardous drinking between the two samples. Associations were not severely affected by selective non-response.

An underrepresentation of parents who misuse alcohol will most likely underestimate the influence that parental alcohol use has on hazardous drinking among offspring. One should therefore also pay attention to associations of minor strength in studies like this.

Second, alcohol misuse in parents was not defined by the use of diagnostic tools, which may have led this study to inaccurately classify parental alcohol misuse [65]. On the other hand, several studies have found that CAGE as an instrument reliably assesses alcohol problems in the general population [66-68]. It should be noted that CAGE was phrased as lifetime-questions in this study, and thus refer to “ever” and not necessarily “current” alcohol misuse. The uncertainty regards the timing of alcohol misuse should be taken into account when interpreting the results.

In addition we were not able to include in the same model information on alcohol use for both parents. Therefore we could not differentiate the effect of having both parents misusing alcohol from the effect of having one parent misusing alcohol. We also did not have information on whether adolescents with parents who misuse alcohol live in the same household as the misusing parent. Thus we were unable to differentiate between adolescents who live in direct exposure and those raised in another environment. It would also have been desirable to apply multilevel analyses to account for possible dependency in the data, but this was not possible with our sample, as we did not have a common family-level identifier for the whole sample.

#### Strengths

Findings from this large representative study population of adolescents constitute an important supplement to previous studies, especially studies based on clinical samples. Moreover, few previous population-based studies have been able to link information independently reported by parents and their offspring. In contrast to many previous studies that have lacked female participants or had predominantly male representation in their materials, we were able to include both mothers and fathers. By including measures of both maternal and paternal alcohol misuse, and outcome measures from both female and male offspring, we were able to make gender-differentiated analyses and contribute new insights to this complex field of research.

## Conclusion

Parental alcohol misuse is associated with an increased risk for hazardous drinking among offspring. Associations vary with both parental gender and offspring gender. Further research should elucidate possible gender-specific mechanisms that could contribute to these versatile findings. More evidence-based knowledge in this field is of great importance for both preventive and clinical practice among parents and adolescents who have a hazardous alcohol consumption pattern.

## Endnotes

^a^(http://www.ntnu.no/hunt/english/data/que Retrieved 18.3.2013).

^b^The question of CAGE applied in HUNT was phrased somewhat differently from the original: “Has someone ever criticized your drinking?”.

## Competing interests

The authors declare that they have no competing interests.

## Authors’ contributions

SHH and GHB contributed significantly in all parts of the research process. TLH is the PI of the Young-HUNT Study. All co-authors have critically reviewed the process and provided suggestions for manuscript revisions. They have all read and approved the submitted manuscript.

## Pre-publication history

The pre-publication history for this paper can be accessed here:

http://www.biomedcentral.com/1471-2458/13/1140/prepub

## Supplementary Material

Additional file 1 **Supplementary material (Tables S5-S7).** Table S5. Characteristics of included versus excluded participants from the Young-HUNT3 survey. Table S6. The multivariate adjusted odds ratios of hazardous drinking in girls by each of the independent and dependent variables included in the multivariate models (Tables 3 and 4). Table S7. The multivariate adjusted odds ratios of hazardous drinking in boys by each of the independent and dependent variables included in the multivariate model (Tables 3 and 4). Click here for file
