# Supplementary material for: Parental alcohol misuse and hazardous drinking among offspring in a general teenage population: gender-specific findings from the Young-HUNT 3 study
Source: BMC Public Health. 2013 Dec 6;13:1140. doi: 10.1186/1471-2458-13-1140 (PMC3866523; doi:10.1186/1471-2458-13-1140)
Supplement: Additional file 1 — Supplementary material (Tables S5-S7). Table S5. Characteristics of included versus excluded participants from the Young-HUNT3 survey. Table S6. The multivariate adjusted odds ratios of hazardous drinking in girls by each of the independent and dependent variables included in the multivariate models (Tables 3 and 4). Table S7. The multivariate adjusted odds ratios of hazardous drinking in boys by each of the independent and dependent variables included in the multivariate model (Tables 3 and 4). [file 1471-2458-13-1140-S1.docx]

### Appendix

**Supplementary material (Tables 5–7)**

**Table 5**. Characteristics of included^a^ versus excluded participants from the YoungHUNT3 survey.

|  |  | **Included**  **(n=5032 )** | | **Not included**  **(n =3168 )** | |
| --- | --- | --- | --- | --- | --- |
|  |  | **%** | **Mean** | **%** | **Mean** |
| **Adolescent age*** | |  | 15.9 |  | 15.8 |
| **Boys** |  | 49.6 |  | 49.8 |  |
| **Girls** |  | 50.4 |  | 50.2 |  |
| **Parents divorced/separated***** | | 24.4 |  | 35.5 |  |
| **High alcohol consumption** | | 20.1 |  | 19.9 |  |
| **Frequent drinking** | | 11.3 |  | 11.6 |  |
| **Frequent intoxication** | | 29.0 |  | 28.7 |  |
| **Hazardous drinking, moderate** | | 17.5 |  | 18.1 |  |
| **Hazardous drinking, high** | | 18.7 |  | 18.4 |  |

^a^ Inclusion criteria: Young-HUNT 3 participants with either mother or father participating in HUNT 3, q1

Information about parents that not participated was not available for comparisons.

*p < 0.05

***p < 0.001

**Table 6**. The multivariate adjusted odds ratios of hazardous drinking in girls by each of the independent and dependent variables included in the multivariate models (Tables 3 and 4)

|  | **High alcohol consumption** | | **Frequent intoxication** | | **Frequent drinking** | | **Hazardous drinking, moderate** | | **Hazardous drinking, high** | |
| --- | --- | --- | --- | --- | --- | --- | --- | --- | --- | --- |
|  | OR | CI 95 % | OR | CI 95 % | OR | CI 95 % | OR | CI 95 % | OR | CI 95 % |
| **Paternal alcohol misuse** | **1.5** | 1.1–2.1 | **1.5** | 1.1–2.1 | 0.9 | 0.5–1.5 | **1.9** | 1.3–2.8 | 1.5 | 1.0–2.2 |
| **Education, father** | 0.8 | 0.8–0.9 | **0.9** | 0.8–1.0 | **0.8** | 0.7–0.9 | 1.0 | 0.9–1.1 | **0.8** | 0.7–0.9 |
| **Marital status** | 1.2 | 0.9–1.7 | 1.1 | 0.8–1.5 | 1.3 | 0.8–2.2 | 0.9 | 0.6–1.4 | 1.3 | 0.9–1.8 |
| **Adolescent age** | **1.2** | 1.1–1.3 | **1.5** | 1.4–1.6 | **1.2** | 1.1–1.3 | **1.7** | 1.5–1.9 | **1.4** | 1.3–1.5 |
| **Maternal alcohol misuse** | 1.3 | 0.8–2.3 | **1.8** | 1.0–3.1 | 1.3 | 0.6–2.9 | **1.4** | 0.8–2.7 | 1.5 | 0.8–2.7 |
| **Education, mother** | 0.8 | 0.8–0.9 | 1.0 | 0.9–1.0 | **0.8** | 0.7–0.9 | 1.0 | 0.9–1.1 | **0.8** | 0.8–0.9 |
| **Marital status** | **1.5** | 1.2–2.0 | **1.4** | 1.1–1.8 | **1.6** | 1.1–2.3 | 1.0 | 0.7–1.4 | **1.7** | 1.3–2.3 |
| **Adolescent age** | **1.3** | 1.2–1.3 | 1.5 | 1.4–1.6 | **1.2** | 1.1–1.3 | **1.6** | 1.5–1.8 | **1.4** | 1.3–1.6 |

Figures in bold are significant at p < 0.05

**Table 7**. The multivariate adjusted odds ratios of hazardous drinking in boys by each of the independent and dependent variables included in the multivariate model (Tables 3 and 4)

|  | **High alcohol consumption** | | **Frequent intoxication** | | **Frequent drinking** | | **Hazardous drinking, moderate** | | **Hazardous drinking, high** | |
| --- | --- | --- | --- | --- | --- | --- | --- | --- | --- | --- |
|  | OR | CI 95 % | OR | CI 95 % | OR | CI 95 % | OR | CI 95 % | OR | CI 95 % |
| **Paternal alcohol misuse** | **1.6** | 1.1–2.3 | 1.4 | 1.0–2.1 | **1.6** | 1.0–2.5 | 1.3 | 0.8–2.1 | **1.7** | 1.1–2.5 |
| **Education, father** | **0.9** | 0.8–0.9 | **0.9** | 0.8–1.0 | 0.9 | 0.8–1.0 | 0.9 | 0.8–1.0 | **0.9** | 0.8–1.0 |
| **Marital status** | **1.5** | 1.1–2.1 | **1.7** | 1.2–2.4 | **2.2** | 1.5–2.1 | 1.1 | 0.7–1.7 | **1.9** | 1.3–2.8 |
| **Adolescent age** | **1.2** | 1.1–1.3 | **1.5** | 1.4–1.6 | **1.2** | 1.1–1.4 | **1.5** | 1.4–1.7 | **1.5** | 1.3–1.6 |
| **Maternal alcohol misuse** | **0.2** | 0.1–0.6 | 0.6 | 0.3–1.1 | 1.0 | 0.4–2.3 | 0.5 | 0.2–1.2 | 0.5 | 0.2–1.1 |
| **Education, mother** | **0.9** | 0.8–0.9 | 0.9 | 0.9–1.0 | **0.9** | 0.8–1.0 | 0.9 | 0.9–1.0 | **0.9** | 0.8–1.0 |
| **Marital status** | **1.6** | 1.2–2.2 | 1.6 | 1.2–2.1 | 1.3 | 0.9–1.8 | **1.6** | 1.1–2.2 | **1.7** | 1.2–2.3 |
| **Adolescent age** | **1.2** | 1.1–1.3 | 1.5 | 1.4–1.6 | 1.3 | 1.2–1.4 | **1.5** | 1.4–1.7 | **1.4** | 1.4–1.6 |

Figures in bold is significant at p <0.05
